# Enhancing Precision: Proposed Revision of FDI's 2-Digit Dental Numbering System

**DOI:** 10.1016/j.identj.2023.12.001

**Published:** 2024-02-12

**Authors:** Arvind Babu Rajendra Santosh, Thaon Jones

**Affiliations:** a School of Dentistry, Faculty of Medical Sciences, The University of the West Indies, Jamaica; b Department of Oral Medicine, Faculty of Dental Medicine, Universitas Airlangga, Surabaya, Indonesia

Sir,

I am writing this correspondence to express my opinion on FDI's 2-digit dental numbering system. Accurate tooth identification within the field of dentistry is of paramount importance. Dental professionals, researchers, and students rely on a standardised system to precisely designate each tooth in the oral cavity. Misdiagnosis due to incorrect notation can result in mistreatment of teeth, ultimately leading to compromised patient care. Furthermore, the misinterpretation of the numbering system can hamper effective communication between dental professionals, leading to misunderstandings, inefficiencies, and even the risk of compromising patient safety.

The FDI 2-digit notation, commonly known as the FDI World Dental Numbering System, is a well-established method for this purpose and used throughout the world. The FDI 2-digit system enhances accuracy in distinguishing between the right and left sides of the mouth as well as between upper and lower dental arches. The significance of a robust tooth numbering system cannot be overstated. It serves as the cornerstone of patient records, treatment planning, dental education, and research endeavours. Precise notation is pivotal in distinguishing between primary and permanent teeth, differentiating quadrants, and specifying individual teeth. Accurate notation is not merely a matter of convenience; it directly influences patient care, treatment outcomes, and the integrity of dental research.

In today's globalised world, dental professionals trained across different regions play a significant role in dental education and clinical training. The FDI tooth numbering system is widely accepted and utilised worldwide, except in the US, where the American Dental Association (ADA) system (1-32, A-T) is predominantly used. This divergence in tooth numbering systems can lead to confusion, especially in dental institutions where educators and clinicians are familiar with both the ADA and FDI systems. This dual approach sometimes challenges the beginner or student dentist. To mitigate this potential source of confusion, the proposal to introduce a dot between numbers in the FDI system aims to clearly differentiate between the 2 numbering systems. The addition of a dot offers a simple yet effective solution to prevent misinterpretation and ensures that tooth identification remains unambiguous and consistent across dental education and practice.

For example, the FDI notation for the upper right first molar is recorded as 16 but pronounced as “one-six.” However, in written form, it appears as 16, which may be read as “sixteen,” resulting in potential misinterpretation with universal system. To mitigate this confusion, we propose incorporating a dot between the 2 digits in notations, for example, 1.6. This adjustment eliminates ambiguity and enhances precision. In contrast, the ADA system numbers teeth from 1 to 32, with 16 referring to the upper left third molar. We advocate for this revision to enhance clarity, and this simple addition of a dot in the 2-digit system can accomplish that (See the Table and Figures 1 and 2).

**Table:** First- and second-digit information in the FDI notation system for primary and permanent dentition.

Permanent dentition	
First digit	Second digit
The upper right is Quadrant #1	Central incisor: 1
The upper left is Quadrant #2	Lateral incisor: 2
The lower left is Quadrant #3	Canine (cuspid): 3
The lower right is Quadrant #4	First premolar: 4
	Second premolar: 5
	First molar: 6
	Second molar: 7
	Third molar: 8


Primary dentition	
First digit	Second digit
The upper right is Quadrant #5	Central incisor: 1
The upper left is Quadrant #6	Lateral incisor: 2
The lower left is Quadrant #7	Canine (cuspid): 3
The lower right is Quadrant #8	First molar: 4
	Second molar: 5

**Fig. 1:**
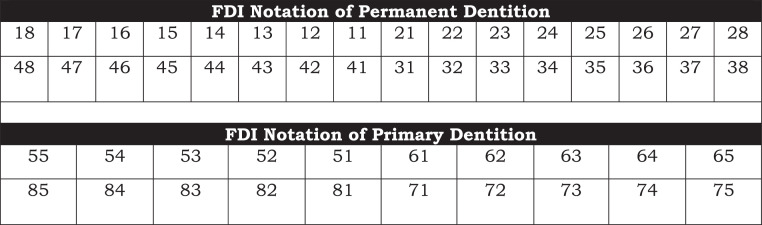
FDI's notation system for primary and permanent dentition.

**Fig. 2:**
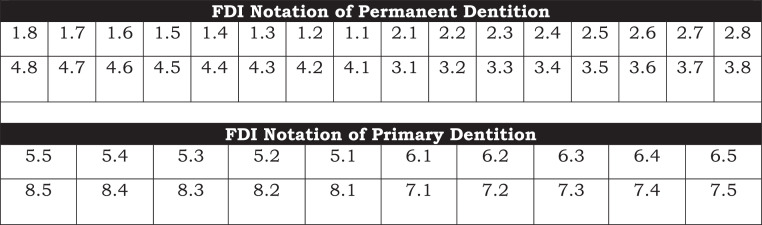
Proposed revisions to FDI's notation system for primary and permanent dentition.

To conclude, the existing FDI notation system has served as a valuable global standard, this proposed revision represents a minor but meaningful improvement that aligns with the ongoing pursuit of precision and clarity in dental notation. This adjustment addresses the issue of similar numerical representations for different teeth within the system, thus reducing the likelihood of misinterpretation and ensuring accurate communication amongst dental professionals globally. It is our hope that this adjustment will be considered for adoption within the dental community to further enhance the accuracy of tooth identification and documentation. This proposed revision can significantly contribute to enhanced clarity and precision in dental communication and diagnosis and prevention of wrong treatment.

## Conflict of interest

None disclosed.

